# Improving home-like environments in long-term care units: an exploratory mixed-method study

**DOI:** 10.1038/s41598-024-62328-0

**Published:** 2024-06-09

**Authors:** Suyeon Bae, Dayoung Kim

**Affiliations:** Department of Housing and Interior Design, Age Tech-Convergence Major, Kyung Hee University, Seoul, South Korea

**Keywords:** Long-term care units, Older adults, Home-like, Mood, Sleep, Environmental social sciences, Health care

## Abstract

Although the number of older adults requiring care is rapidly increasing, nursing homes have long faced issues such as the absence of a home-like environment. This exploratory mixed-method study investigated how residents (n = 15) in a long-term care unit in South Korea perceive home-like features and privacy in their living spaces. The results indicated that most participants were satisfied with the homeliness and privacy of their environment, but some were unhappy with the level of privacy. Most participants had low scores on the Geriatric Depression Scale and the Pittsburgh Sleep Quality Index, indicating low levels of depression and sleep disorders. Sleep quality was affected by factors such as sensory environment, staff visits, and room temperature. Although participants appreciated social support and private rooms, they expressed a desire for larger rooms. Overall, this study provides preliminary insights into older adults’ views on their living spaces in long-term care with implications for improving their quality of life.

## Introduction

Due to the global phenomenon of population aging, the number of older adults requiring care is increasing. The demographic age dependency ratio for those 65 years and older is projected to rise from 31 to 47% by 2050 in developed nations^1^. Consequently, there is a growing demand for more senior-assisted facilities such as long-term care units (LTCUs).

Nursing homes have long faced issues such as the absence of a home-like environment and insufficient staffing; the latter, in particular, was exacerbated during the COVID-19 pandemic^2^. Moreover, many older adults prefer to remain in their homes as long as they can. Specifically, the desire for deinstitutionalization has intensified in the United States amid the COVID-19 crisis^2^.

“Home” holds unique significance, embodying various meanings for each individual^3^. A nursing home plays a dual role as a clinical care facility and a communal living space to support a fulfilling life^3^. Moreover, it is crucial to deliver high-quality care within environments that resemble a home^4^. Studies have shown that residents in such environments experience higher levels of satisfaction, comfort, and a sense of belonging compared with those in more institutionalized settings^5^. Additionally, home-like environments are conducive to social interactions and community engagement among residents. Communal spaces, such as living rooms and dining areas designed to resemble those found in private homes, encourage residents to socialize, participate in activities, and form meaningful connections with their peers^6^.

Numerous empirical studies have identified that the physical environment, social environment, neighborly relations, daily life routines, and quality of care are key elements that contribute to the homelike feel of a nursing home. Despite being distinct, these elements interact and affect one another^7^. A comprehensive literature review using the snowballing method revealed that environmental factors such as private spaces, personal items, atmosphere, optimal stimulation levels, nature connectivity, and social engagement are crucial for fostering a sense of home^8^. Specifically, private spaces allow residents to personalize and control their environment, host guests individually, and maintain privacy through private bathrooms^9^.

This study employs a mixed methods approach to address these questions and comprehensively capture the complexity of older adults’ experiences in LTCUs in Seoul, South Korea. By integrating quantitative and qualitative data collection methods, this study aims to enrich our understanding of the multifaceted factors influencing residents’ perceptions of their living spaces. Specifically, we utilize a sequential explanatory mixed methods design, whereby quantitative data on mood and sleep quality will be initially collected, followed by qualitative interviews to delve deeper into participants’ perceptions of home-like features and privacy conditions. This approach allows us not only to quantify the prevalence of certain experiences but also to explore the underlying reasons and nuances behind these experiences. Combining quantitative and qualitative data enables a more nuanced and holistic analysis, providing valuable insights to inform the development of interventions and improvements in long-term care environments.

## Literature review

Creating a home-like environment includes both tangible and intangible elements^10^. Although some research in this area tends to be normative and difficult to generalize, establishing methods for fostering a sense of home is crucial. This can involve incorporating elements such as home furnishings, sounds, scents, and visuals that evoke residents’ past experiences and contribute to a homely feeling in their current living space. However, the focus should not be solely on environment creations but also on practical applications^10^. Home-like features are pivotal in fostering a sense of comfort and familiarity for residents in long-term care settings. Research suggests that incorporating elements such as familiar interiors, personalized belongings, and outdoor spaces can enhance residents’ overall well-being and satisfaction with their living environment^11^. Moreover, the creation of homely atmospheres has been associated with reduced feelings of social isolation and increased engagement in meaningful activities among residents^12^.

Lee et al.^11^ outlined key features for fostering a home-like atmosphere, emphasizing the importance of familiar interiors, such as wallpaper and traditional furniture, which evoke memories and foster feelings of connection for residents. These elements serve as a vital link between past and present lives, enhancing residents’ sense of identity. Personal items, such as photos and belongings, carry emotional significance and contribute to personalizing and organizing living spaces^9^. Photographs, in particular, can reinforce connections with family and friends, providing a sense of belonging and continuity^9^. However, restrictions on space or personal items because of facility rules can infringe on residents’ autonomy, leading to feelings of alienation and stress in an unfamiliar environment^9^. It is also recommended that such personal elements be placed within residents’’ daily paths, ensuring they are easily accessible with staff assistance^8^. Furthermore, limited access to gardens and outdoor areas and the inability to visit these spaces independently can negatively affect health and wellbeing^8^. Finally, involving residents in the enhancement of nursing home environments can bolster their emotional connection, sense of ownership, and autonomy within their living spaces^8^.

Nursing home residents often prioritize their privacy, starting with bathroom use, where they wish to carry out hygiene routines without intrusion^13^. Privacy refers to an individual’s right of individuals to control access to themselves and their personal information, actions, and spaces^14^. It encompasses various aspects such as confidentiality, autonomy, and the ability to maintain boundaries. In the context of long-term care facilities, privacy entails the privacy of personal information, medical consultations, and personal care activities, ensuring that residents feel a sense of respect and dignity^15^. Regarding visual privacy, studies have highlighted the importance of visual privacy in enhancing residents’ sense of dignity and autonomy, as well as reducing feelings of vulnerability and discomfort^16^. Acoustic privacy pertains to controlling the transmission of sound within the environment. Residents in LTCUs desire a quiet environment; while they understand the communal nature of nursing homes, residents still seek a peaceful, noise-free setting^13^.

Personal space refers to the physical area surrounding an individual that they consider their own and where they feel comfortable and secure^17^. It includes both physical boundaries, such as the space around one’s body, and psychological boundaries, such as the need for autonomy and control over one’s environment. In long-term care settings, personal space can manifest in the form of private rooms or areas designated for individual activities, providing residents a sense of ownership and control over their living environment^18^. Overstepping the boundaries of an individual’s tolerable space can lead to conflict, tension, and discomfort, underscoring the need for private living spaces^19^. Furthermore, research on nursing home design indicates that providing personal space significantly reduces anxiety and aggression, minimizes resident conflicts, decreases psychiatric medication use, and enhances sleep quality^19^. Residents in larger facilities, where living quarters are often delineated by mere curtains, frequently express concern about insufficient privacy because of the lack of soundproofing and visual barriers^13^.

Mood and depression are significant determinants of residents’ quality of life in long-term care settings. The transition to communal living environments can evoke feelings of loneliness and social disconnectedness, exacerbating preexisting mood disorders among older adults^20^. Therefore, implementing interventions aimed at enhancing social support networks, promoting leisure activities, and providing access to mental health services is crucial for addressing residents’ emotional needs and reducing the prevalence of depression within long-term care facilities^21^.

Sleep quality emerges as a key indicator of residents’ overall health and well-being in long-term care settings. Factors such as ambient noise levels, lighting conditions, and room temperature can significantly impact residents’ sleep patterns and quality of rest^22^. By prioritizing the creation of quiet, comfortable sleeping environments and offering personalized sleep hygiene interventions, long-term care facilities can support residents in achieving restorative sleep and maintaining optimal physical and cognitive functioning^19^.

Most studies included in the literature review were conducted in Northern Europe, Australia, New Zealand, and North America. Given the cultural differences, it is challenging to generalize their findings to other countries. Owing to limited space in urban areas, LTCUs in South Korea may prioritize compact design elements to maximize efficiency without compromising quality of care. The minimum for each room should be 16 m^2^ (171 ft^2^). This could involve innovative use of space-saving furniture and multi-functional rooms. Moreover, akin to other countries, buildings must adhere to safety regulations, including fire safety, emergency exits, and accessibility for residents with mobility impairments.

Culturally, a significant aspect in South Korea is the emphasis on familial involvement and filial piety, where family members often play a central role in caregiving and decision-making for older adults. This familial support extends to the caregiving environment, where spaces are designed to accommodate family visits and activities. Additionally, Korean culture emphasizes on communal living and social connections, leading to the integration of shared spaces and group activities within long-term care facilities to foster a sense of community and belonging among residents. Therefore, researching LTCUs in South Korea from a cultural perspective is vital for tailoring care practices and environments to align with Korean cultural norms and values.

An explorative study aimed to investigate the following research questions:

1. What are older adults’ perceptions of their living spaces in LTCUs, specifically in terms of home-like features of visual and acoustic privacy?
2. How do these perceptions relate to their mood?
3. How do these perceptions relate to their sleep quality?
4. Which home-like features in LTCUs enhance their privacy, mood, and sleep quality?

## Materials and methods

### Settings

**Figure 1:**
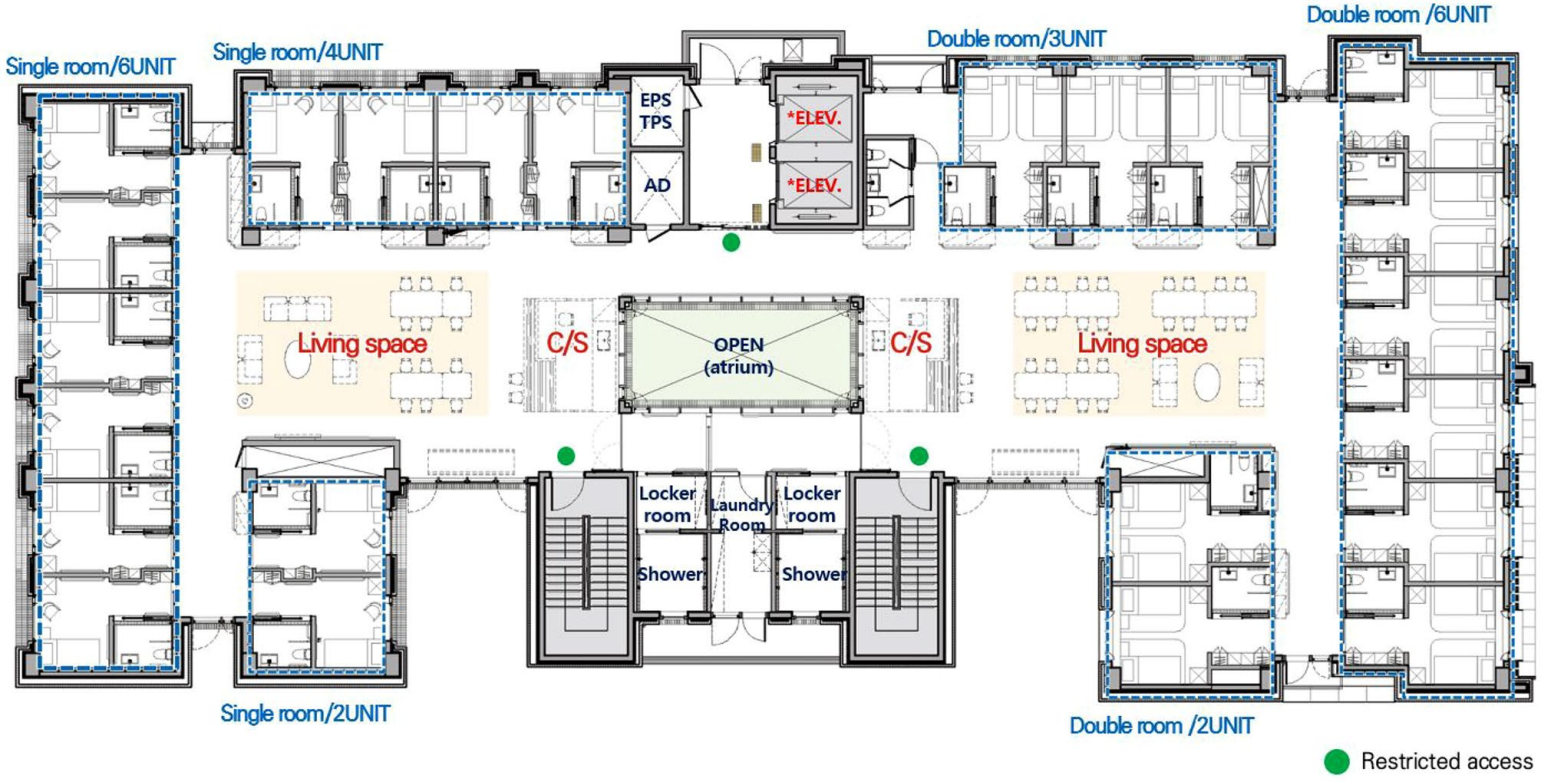
Overall floor plan showing the residential units and shared spaces. (Source: KB Goldenlife)

**Figure 2:**
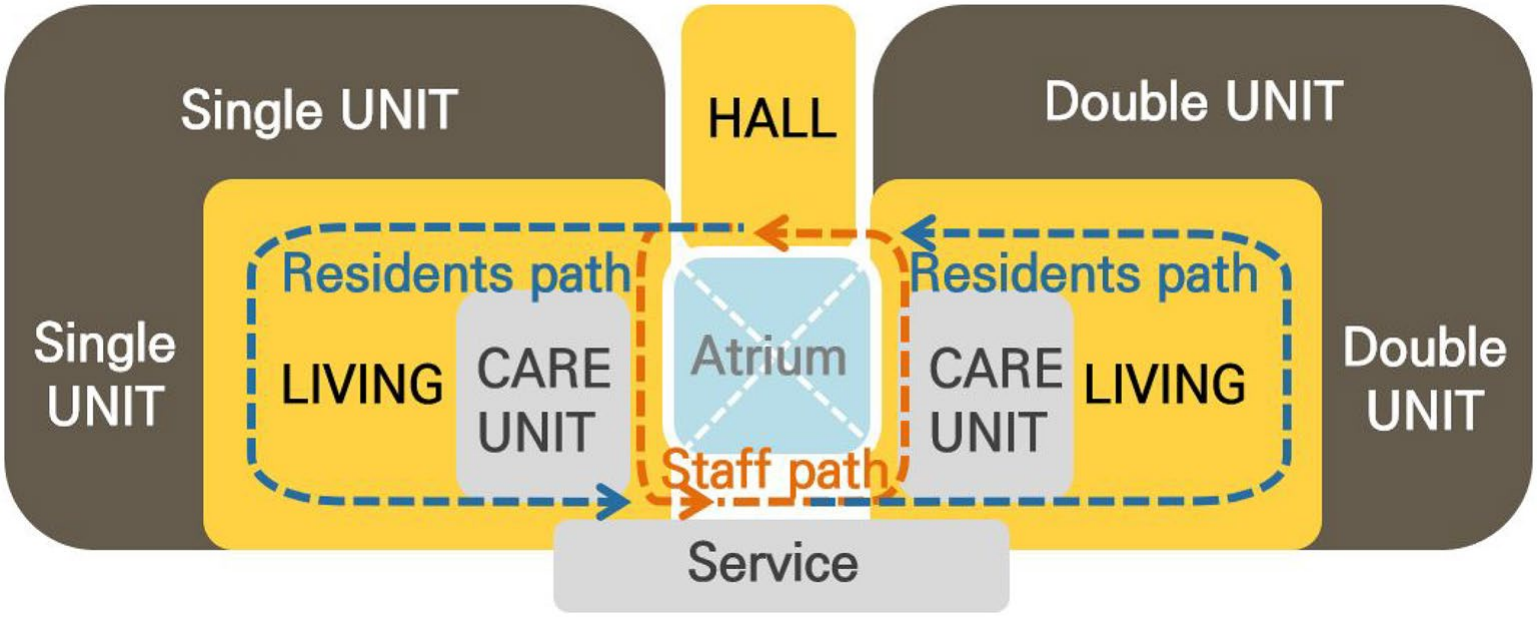
Diagram showing the core, paths, residential units, and shared spaces. ﻿(Source: KB Goldenlife)

**Figure 3:**
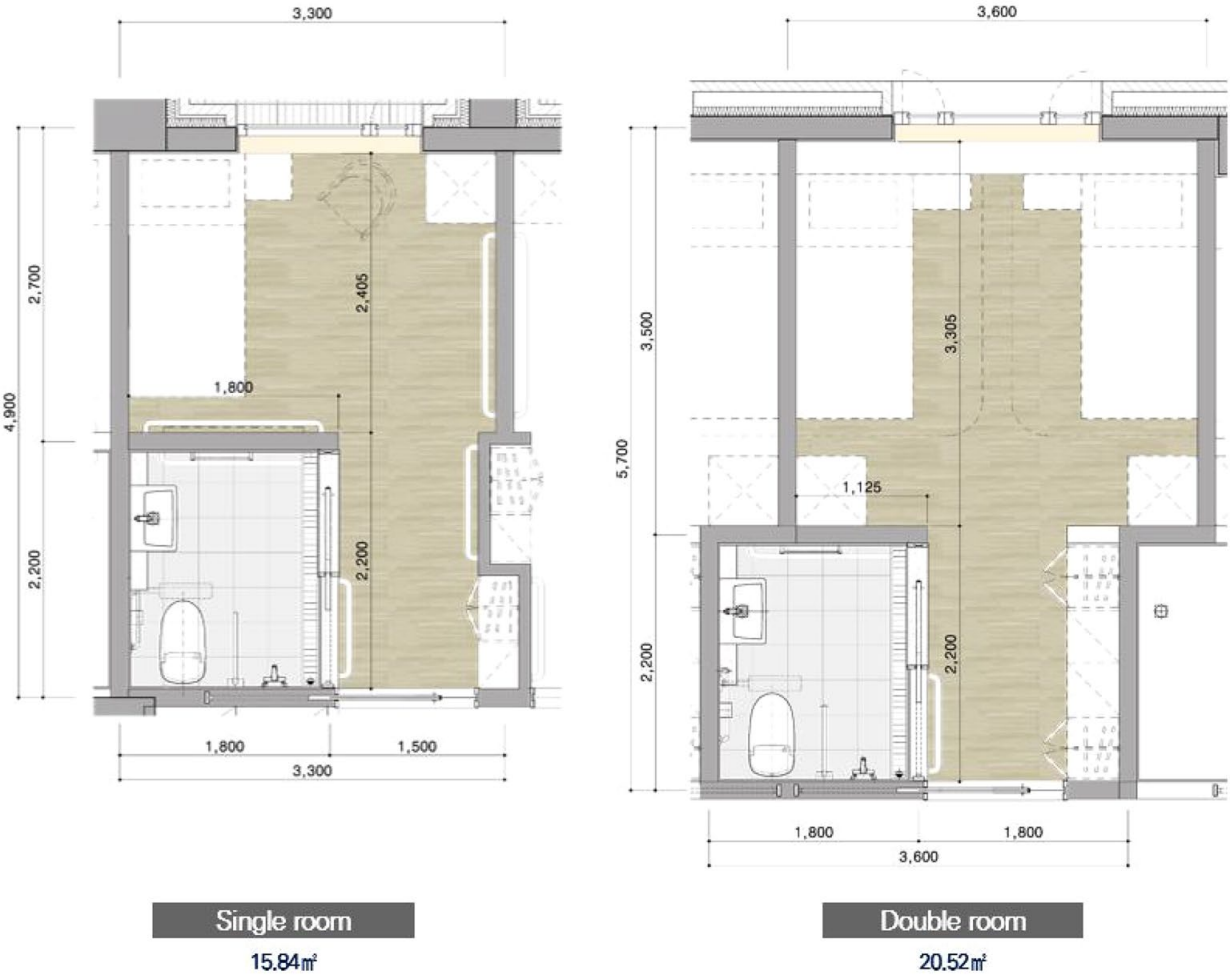
Detailed floorplan for the single room and double room. ﻿(Source: KB Goldenlife)

The study was presented to the recruited LTCU, where participants expressed their willingness to participate. The facility is a three-story building with 36 single rooms and 22 double rooms. The single rooms are evenly distributed across the first floor to the third floor, while the double rooms are located on the second and third floors. Figure 1 presents the floorplan of the second/third floor and Fig. 2 shows a diagram showing the core, paths, residential units, and shared spaces. Staff paths include residents’ paths. Both private and double rooms have their own bathroom, consisting of a toilet, a basin, and a shower (see Fig. 3).

### Participants

Facility staff screened volunteers to ensure they could communicate effectively and were free of cognitive disorders in addition to having resided in the facility for at least six months. Of 80 participants, 15 participants were interviewed (see Table 1). Most participants were women (n = 11, 73%), with an average age of 87.2 years. Of these, eight participants had roommates in double-occupancy rooms, whereas seven lived in a private rooms.

**Table 1:** Demographic information about the participants.

Participants	Gender	Age	Roommate
1	M	92	Y
2	F	88	Y
3	F	92	N
4	M	92	N
5	M	88	N
6	F	84	Y
7	F	88	Y
8	F	80	Y
9	M	88	N
10	F	92	N
11	F	83	N
12	F	85	N
13	F	88	Y
14	F	85	Y
15	F	83	Y
Mean (SD)	–	87.2 (3.80)	–

The decision regarding the sample size for this study was influenced by practical considerations and the nature of the research context. Given the limited pool of eligible participants within one single LTCU where the study was conducted, the sample size was determined by the number of individuals who met the inclusion criteria and expressed willingness to participate. No formal power analysis was performed; however, the sample size of 15 participants was deemed appropriate to capture a diverse range of experiences and perspectives within the study population. Although efforts were made to ensure data saturation through iterative data collection and analysis, the small sample size may limit the generalizability of the findings, particularly in the context of a triangulation design. It is important to acknowledge these limitations and interpret the results within this context.

### Procedure and measurements

The research team tried to establish a comfortable environment for the participants and started with informal conversations to build rapport prior to the interviews. The interviews were conducted in residents’ rooms and enclosed spaces for privacy. The interview questions used straightforward and non-technical language to facilitate a better understanding for older adult participants to aid in answering the questions. The questions were reviewed by the staff members. Additionally, the interviews were conducted at a pace that allowed participants to proceed and respond comfortably.

The interview began by gathering demographic data, including participants’ gender and age. It focused on four key areas: mood, sleep quality, home-like features, and privacy. To assess mood and sleep quality, we used the translated Geriatric Depression Scale (GDS) short form, consisting of 15 questions, and the translated Pittsburgh Sleep Quality Index (PSQI), respectively. The GDS short version comprises 15 yes/no questions designed for older adults. A score ranging from 0 to 5 indicates no presence of depression, that from 5 to 10 suggests mild depression, and a score from 10 to 15 indicates severe depression^23^. The inventory has good reliability (*r* = 0.85), and a high level of validity, ranging from 0.83 to 0.84^24^. The PSQI is developed to measure sleep quality, including duration of sleep, wake patterns, sleep-related problems, and so on^25^. The PSQI, being the most commonly utilized assessment tool for sleep quality, has been translated into 48 languages, making it widely accessible^25^. The PSQI has a high internal consistency (*r* = 0.83) as well as a high test–retest reliability^26^.

Although the GDS and PSQI are written measurements, we verbally administered the questions. Given the participants’ average age, they might struggle to read, understand, and answer the questions; thus, the research team verbally asked the questions and gave further explanations if needed. We also explored participants’ perceptions of home-like features and privacy in their living spaces. Satisfaction with these aspects was measured using a series of questions for each theme, rated on a five-point Likert scale. In addition to the semi-structured interviews, which served as the primary data collection method, the study employed triangulation to gain deeper insights into participants’ views. Triangulation involves the use of multiple data sources, methods, or researchers to corroborate findings and ensure a comprehensive understanding of the research phenomenon. In this study, triangulation was achieved by incorporating both qualitative and quantitative data collection methods, as well as involving multiple researchers in the data analysis process. The qualitative data obtained from the interviews provided rich insights into participants’ perspectives, whereas quantitative measures such as the GDS and PSQI offered complementary information on mood, sleep quality, and other relevant factors. By triangulating data from different sources and methods, the study aimed to strengthen the credibility and robustness of the findings, thereby enhancing the overall trustworthiness of the research outcomes. Additionally, we included open-ended questions about home-like features and overall privacy, including visual and acoustic privacy (see Table 2).

**Table 2:** Interview measurements and questions used.

Topics	Questions
Depression	GDS Short Version (15 questions)
Sleep quality	PSQI
Home-like features	1. How satisfied are you with the home-like features here? If you express satisfaction from 1 (very dissatisfied) to 5 (very satisfied), how satisfied are you? 2. Would you please share the reason for satisfaction/dissatisfaction? 3. What would you like to improve here for a more home-like environment?
Privacy	1. How satisfied are you with the overall privacy here? If you express satisfaction from 1 (very dissatisfied) to 5 (very satisfied), how satisfied are you? 2. Would you please share the reason for satisfaction/dissatisfaction? 3. How about visual privacy? Are you satisfied or dissatisfied? 4. Would you please share the reason for satisfaction/dissatisfaction? 5. What would you like to improve here for better visual privacy? 6. How about acoustic privacy? Are you satisfied or dissatisfied? 7. Would you please share the reason for satisfaction/dissatisfaction? 8. What would you like to improve here for better acoustic privacy?

### Ethical approval

This study was approved by the university institutional review board (IRB) of Kyung Hee University [KHSIRB-22-160]. Participants provided verbal consent to participate in the study after being read a consent form, as approved by the IRB. The interviews were conducted in person and lasted between 30 and 45 min. The audio of the interviews, including verbal consent, was recorded. All methods were performed in accordance with the approved guidelines and regulations by the IRB.

### Data analysis

The recorded interviews were transcribed using the services of a dedicated company specializing in transcription. Qualitative data analysis was conducted using thematic analysis, a method commonly employed in qualitative research to identify patterns and themes within the data. Two independent researchers performed the analysis manually, initially familiarizing themselves with the data to develop preliminary themes based on the theory of supportive design^27^. The theory of supportive design focuses on creating environments that support the physical, emotional, and social well-being of individuals. These themes were refined through iterative reviews and discussions to ensure comprehensive data coverage. While a formal codebook was not developed, the researchers independently coded the responses into primary and secondary themes using Microsoft Excel. This process facilitated the identification of central themes and patterns within the data. To enhance the reliability of the coding process, the researchers compared their coding and resolved any discrepancies through consensus. The inter-rater reliability was calculated as the ratio of agreements between two independent researchers, indicating the level of consistency in their coding or data. The inter-rater reliability coefficient for this process was calculated to be 0.92, indicating a high level of agreement between the researchers. While qualitative software tools were not utilized in this study, the manual approach enabled a thorough and systematic analysis of the data.

## Results

Table 3 summarizes the quantitative data. Of a possible 15 points, most participants had low scores, ranging from 2 to 7, regarding their mood. Only two participants (P2 and P3) received a score of seven. The maximum score for sleep quality was 24 points, with higher scores indicating poorer sleep quality. Participant P3 scored the highest with 21 points, whereas P4 had the lowest score of 0 points. The average satisfaction scores for the home-like atmosphere and privacy conditions suggest that most participants were content with these aspects. However, participants P2 and P3 expressed extreme dissatisfaction with their level of privacy.

**Table 3:** Participant demographic information.

Participants	GDS	PSQI	Homelike	Privacy
1	5	2	5	5
2	7	10	4	2
3	7	21	3	1
4	4	0	5	5
5	4	8	4	5
6	4	15	4	5
7	5	12	5	5
8	2	6	4.5	3.7
9	3	2	5	5
10	4	15	5	5
11	3	3	5	5
12	6	12	4	3.7
13	4	4	4	4.3
14	4	16	4.5	5
15	2	8	3.5	4.3
Mean (SD)	4.27 (1.53)	8.93 (6.18)	4.37 (0.64)	4.27 (1.23)

Homelike and Privacy are measured on a 5-point Likert scale (1 indicates “very dissatisfied”; 5 indicates “very satisfied”).

*GDS* geriatric depression scale, *PSQI* pittsburgh sleep quality index, *SD* standard deviation.

### Quality of sleep

The open-ended question responses highlighted specific factors affecting participants’ sleep quality beyond their PSQI scores (see Table 3). Those with scores above 12 perceived their sleep as poor. Two participants (P5 and P8) reported poor sleep despite having moderate PSQI scores.

#### Sleep disruptors

Participants identified various factors affecting their sleep quality, with noise, light, and temperature being the most cited disruptors (see Table 4). Comments regarding noise disturbances were frequent, with 7 of 15 participants mentioning noise from hallway traffic, staff activities, or other residents as interfering with their ability to sleep. Light disturbances were also prevalent, with three participants commenting on the brightness of nighttime health checks or hallway lighting disrupting their rest. Temperature discomfort was noted by two participants, particularly regarding overheated rooms affecting their ability to sleep. P3 mentioned that she was unable to adjust her blanket to cool down because of her health condition.

**Table 4:** Primary themes and subthemes for sleep quality and selected quotations.

Primary theme	Subtheme	Participant	Quotation
Sensory environment disruption	Noise	Participants frequently mentioned disturbances from various sources such as staff, other residents, and hallway traffic	
		P8	This old lady (my roommate) talks a lot, like she’s talking to herself. That’s one thing, but everything else is quiet
		P12	I’m just surprised by someone who is screaming here
		P13	I have to wake up at dawn when my roommate makes sounds
	Light	Some participants reported disruptions owing to staff conducting nighttime health checks, which involved turning on lights	
		P8	I don’t usually nap, so I just try to get a good night’s sleep. I don’t like lights when I try to sleep. I don’t like electric lights I just come in after dinner, close the curtains, turn off all the lights
			If I press the call and the staff member comes and takes care of me, it’s bright again If she takes care of them, then I cannot sleep right away. Because I saw the light, and because of the care, and because the staff came in and out, I think…
	Room temperature	Uncomfortable beds were identified as a factor affecting sleep, with participants experiencing difficulty sleeping or waking up due to room temperature	
		P3	When it’s hot, it’s too hot in here It gets unbearable. When I get a cold and I sweat, I can’t take the blanket off myself Even in the summer, I have to adapt to it So that’s why it’s hard for me
			Participants mentioned additional factors such as uncomfortable bedding, various health conditions, anxiety, and the presence of a roommate as contributors to poor sleep quality
Other factors		Participants mentioned additional factors such as uncomfortable bedding, various health conditions, dreams, anxiety, and the presence of a roommate as contributors to poor sleep quality	
		P1	I often have strange dreams sometimes
		P5	I’m a good sleeper. But it’s because of something I care about, I don’t sleep well these days. I’m worried about it, and sometimes my blood pressure is a little high
		P6	I can’t sleep. My bed is uncomfortable. I struggle a lot
		P7	I don’t fall asleep right away, it takes me at least an hour or more to fall asleep And once I wake up, I have a hard time sleeping after that
		P8	I have trouble sleeping when I’m worried and upset about something Otherwise, I just try to go to bed early
		P10	I got my 3rd COVID-19 vaccine shot and developed glaucoma, so the staff have to come in and out frequently. That interferes with my sleep
		P13	My roommate defecates a few times at night, and then I get a headache due to the smell. The smell hurts a lot The smell makes me sick That’s why I keep saying, I can’t stay with my roommate because she smells so bad, although she was a very good person
		P14	I’m not sleeping well because I’m sick. I’m fine except when it hurts too much

#### Psychological factors

Apart from environmental factors, participants also mentioned psychological factors influencing their sleep quality. Anxiety, discomfort, and health conditions were highlighted as contributed to poor sleep among participants. Three older adults expressed feelings of anxiety or discomfort related to their living environment or personal health issues, which affected their ability to fall or stay asleep.

### Home-like environments

#### Social support and physical environments

**Figure 4:**
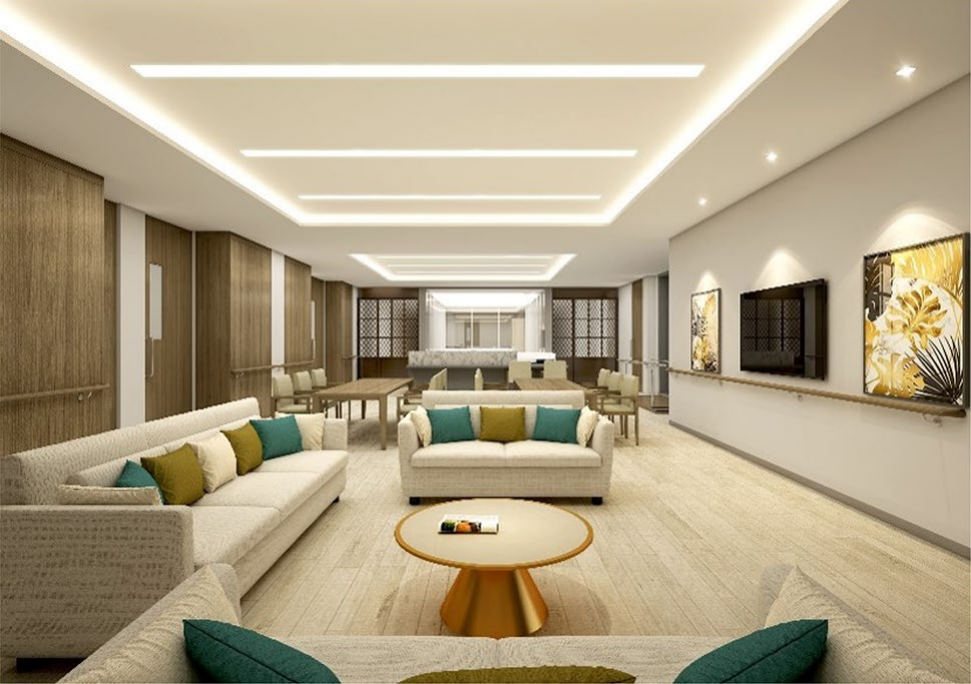
An image of living spaces. ﻿(Source: KB Goldenlife)

Most participants expressed satisfaction with the social support provided by staff and family members within the LTCU (see Table 5). Seven participants felt that the staff’s care, especially when they were unwell or uncomfortable, contributed to a homely atmosphere. Two others valued the opportunity to spend time with family within the facility. Regarding physical environments, the living space design was the second key satisfaction factor, with five participants appreciating the private rooms and distinct areas for the dining room, living room, and bedroom. There are two common living rooms, one in each wing (see Figs. 1 and 2). These living rooms can be directly accessed from all bedrooms. In essence, the living room is positioned at the center of the space. Therefore, when residents exit their bedrooms, they enter the common living room, similar to the layout of a typical house. In addition, the living rooms were designed to create a comfortable and cozy feeling (see Fig. 4). Finally, three participants attributed their sense of home to the facility’s offerings, such as homemade meals and rehabilitation services.

**Table 5:** Primary themes and subthemes for home-like environments and selected quotations.

Primary theme	Subtheme	Participant	Quotation
Factors contributing to home-like atmosphere	Social support	Participants valued social support from staff, family, and the surrounding community, which contributed to a sense of home	
		P1	I’m happy with the facilities here, and my family is often here together
		P3	The staff always take care of me here and they see me when I’m struggling, so it’s nice
		P7	I’ve got helpers here and there, and they’re really nice. They’re kind So, I just do what they tell me to do, so they feed me, they give me something to eat, they give me a snack, they give me a bath, they do everything The most important thing here is that the helpers are very nice. That’s what I like the most
		P8	Actually, there are a lot of programs here and the teachers are all so enthusiastic and helpful, it makes me feel comfortable
		P11	I feel comfortable with everything. Everyone here is great
		P14	I think it’s just the facilities and the people here just maintain human relationships without any difficulties. That’s why I feel comfortable
	Physical environment design	Private rooms, distinct spatial layouts, and offerings such as homemade meals and rehabilitation services were identified as factors contributing to a home-like atmosphere	
		P4	It’s better than the structure, but if you compare regular nursing homes, this is the best one, I think! Scientifically, normally, you walk out the door of your room and there’s nothing. It’s just a hallway, and you go down the hallway, and you gather, and you eat. This place is like 12 rooms in a row, and then you go out and it’s a hall, and then it’s a communal living area, and then the dining room, which is completely separate from the bedrooms. It’s all in one place It’s all together like that, and it’s really nice here
			The bedroom is 16 m^2^ (172 ft^2^). There’s nowhere else that’s so organized and engineered for 16 m^2^ per room If somebody who can’t walk very well sits down, there’s a bed, a table, everything around them, so they can just move a little bit and put everything next to them
		P5	Recreation center, I think it’s the fourth floor now It’s on the fourth floor, it’s a rooftop, and now there’s a recreation center, and there’s equipment and spaces where you can work out on your own So, it’s much better because it prevents you from bending over while exercising So, it’s very helpful for people with physical disabilities
		P6	I’m comfortable because I have my own room
		P7	This is all about scenery There’s nothing special to do here Since they do everything for you, it’s frustrating because you can’t go out as you want It’s not a landscape, but a car going back and forth. I see people going back and forth It relieves my frustration. Since this is a big road
Areas for improvement		Participants expressed dissatisfaction with certain aspects of the living environment, including the desire for larger rooms, personal space, and improved neighborhood relations	
		P2	What I want to change is… I want you to put a television in the double room. Since we don’t have a house, our old guys always lived with television I can’t go outside as I want, and I can take the teachers out, so I can’t take them home… But I can’t be outside all the time to watch TV. It gets uncomfortable… There’s no television in the bedroom as I am in a shared room. I’m a little upset because I can’t watch it in my room
		P4	The degree of health among the residents is very different. There are people who don’t remember well, people who are uncomfortable walking, and people who want to need support going to the toilet… People who walk with a walker It would be better to separate people (based on their health conditions)
		P5	It’s a recreation room I wish there were more recreational facilities I wish there was a recreation center or something like that I mean, I’m a relatively healthy person
		P6	Speaking of spaces I’d change… Group living has its advantages, but it also has its disadvantages It’s too crowded and noisy. There’s no personal space. I need my own space. It’s not an office. Basically, if I could, I would need more personal space If I were allowed to have personal space, which I can’t, I’d like to have a place where I can listen to the radio and think about music in private, because I can’t right now
		P8	Some people don’t care about hygiene Even people with dementia, they spit on the floor, they scrub the floor all the time, and when it dries, where does it go? It goes down our throats So, it’s just a really, really deadly problem And I’m not even supposed to say this, but I’m in a position where I can’t just let it go
		P14	The space (bedroom) is a little too tight
		P15	It’s a bit uncomfortable to take a shower in the bathroom The staff takes me to shower and does all the laundry for me here, but I don’t like how they do laundry, and I’m a little uncomfortable with that. They’re nice to me, though

However, some participants were dissatisfied with social support and physical environments. For example, three participants (P7, P8, and P15) wanted to forge stronger connections with their neighbors. Two participants (P6 and P12) felt that their autonomy was restricted by living in shared spaces. Last, because of the emotional toll of witnessing others in varying health stages, participants advocated for the segregation of residents based on health conditions, seeking living arrangements that cater to individual needs and health statuses.

#### Desires for personal space

**Figure 5:**
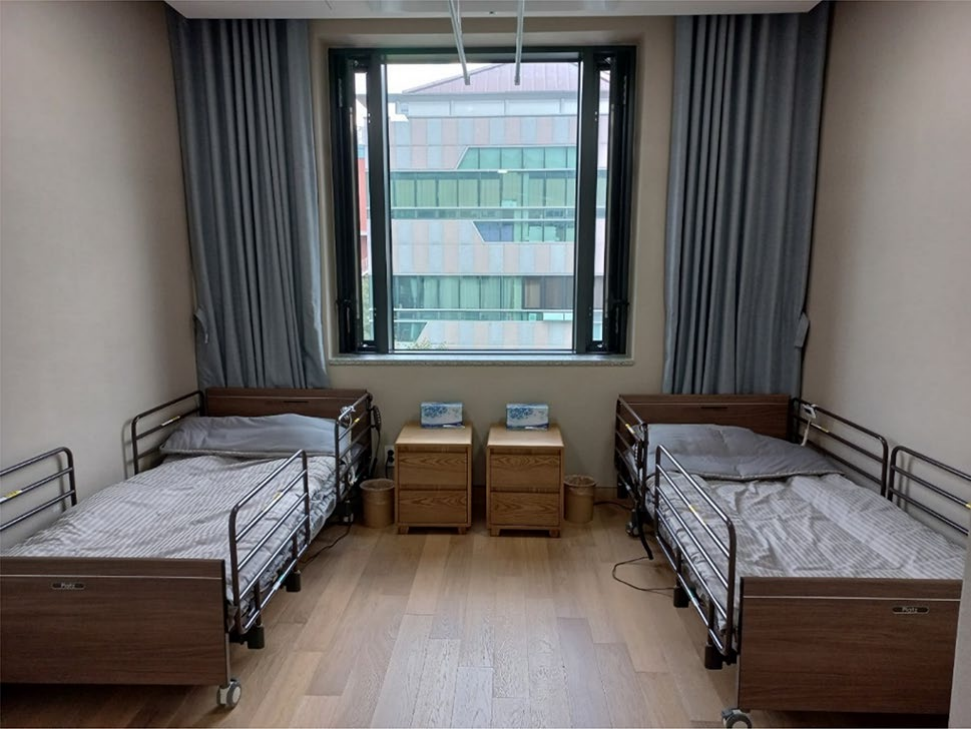
A picture of a double room. ﻿(Source: KB Goldenlife)

Several participants expressed dissatisfaction with the home-like features, providing specific reasons for their discontent. Despite high satisfaction scores on the five-point Likert scale (Table 3), many participants offered suggestions for improvements when prompted about enhancing their home-like environments. Eight participants wanted to enlarge their physical spaces, with P4 appreciating the efficient organization of furniture in his compact room but still wishing for more space. All the necessary elements for living are included in the residents’ rooms, but the area is not spacious, especially for double rooms (see Fig. 5 which shows an unoccupied double room). P6, who has a roommate, said she wished to have her own private space because she could not even play the radio without fear of disturbing her roommate. The absence of televisions (TVs) in the shared rooms led to a common request for personal TVs and clocks.

### Privacy

#### Acoustic and visual privacy

**Figure 6:**
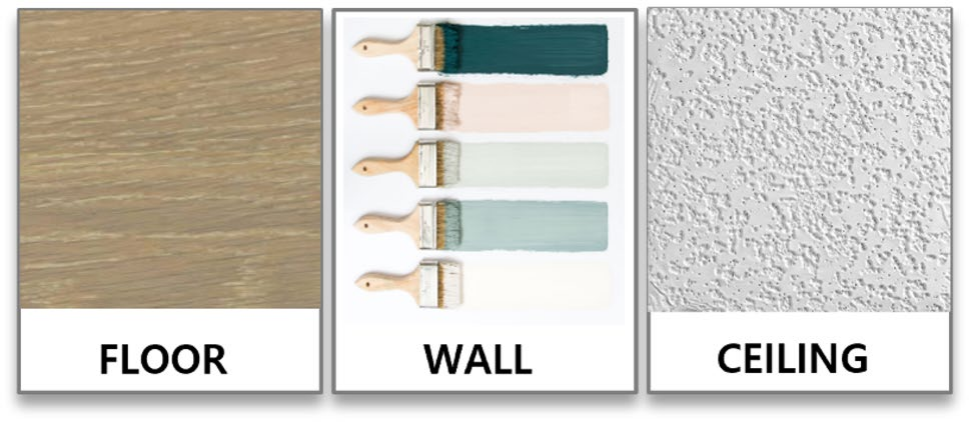
Materials used in residential units. ﻿(Source: KB Goldenlife)

Generally, the participants were satisfied with their privacy conditions (see Table 6). They particularly appreciated the physical aspects of acoustic privacy, such as soundproof doors and walls, which they found highly effective. The floor is made of wood and is effective in soundproofing (see Fig. 6). Participants noted that when the door was closed, noise was significantly reduced. Regarding visual privacy, the design of the space included areas not visible from the hallway, and the combination of a door and curtain provided additional visual privacy.

**Table 6:** Primary themes and subthemes for privacy and selected quotations.

Primary theme	Subtheme	Participant	Quotation
Satisfaction with privacy conditions		Participants generally expressed satisfaction with acoustic and visual privacy, attributing it to factors such as soundproof doors and walls, spatial layout, and privacy features offered by the facility	
	Acoustic privacy	P4	I can barely hear it when I close the door. The soundproofing is good The walls are very thick, so you don’t know what’s going on in the next room If you don’t make a big noise, the soundproofing is good The doors are also soundproofed So it’s the best nursing home I’ve ever been in
		P5	I can’t hear much (sound from outside to inside) It’s completely blocked in here (bedroom) and I can’t even see the hallway outside
		P6	We’re social animals, so we shouldn’t have no sound at all, we should have some sound, because we’re anxious when we don’t have sound. So, this much noise is okay to deal with
		P7	I can’t hear anything outside. I can hear people eating in the dining room, but not outside
		P8	Now, if I leave the door open, the noise from outside comes in, and if I close the door to our room, it’s pretty quiet
	Visual privacy	P4	I had this (recliner), and now I’m sitting here on this one, and I can’t see over there, because there’s a door. So, I can leave the door open, and I can sleep in here by myself anytime I want, and it’s a secret room up here. You can’t see out here very far Even if you leave the door open, you can’t see anyone passing by outside if you sit right here
		P13	But, I can sit here with the curtains like this. I open that door, a lot of people come in. If I close the door, they don’t come in. So, I can handle it
	Overall privacy	P8	My roommate, she’s usually out in the restaurant all day, so I’m kind of relaxed The person next to me (roommate) makes me feel that I am not in a double room in the daytime If you have a two-person room and the person next to you is always coming in, it’s very uncomfortable and not good. But, I feel like that I live in a single room most of the time during the day, so yeah I’m very comfortable
		P10	I’m in my room, so I have a lot of privacy
		P12	My room is the end of the corridor, no one’s coming through here anymore. It’s okay, I’ll just close the door if I want more privacy
		P14	I just think my privacy is quite well protected
Dissatisfaction and Suggestions for Improvement	While overall satisfaction was high, some participants expressed desires for larger rooms, personal TVs, improved soundproofing, and stronger connections with neighbors		
		P2	The view is limited. It’s a little uncomfortable If I go out, then I can see the mountains from where I’m sitting. I can see them all the way out there. But, I can’t see them from here (my room). So, it’s quite uncomfortable in the room I do a lot of things in my room, but I don’t have a TV, I don’t have a clock. That’s uncomfortable
		P3	I can’t get up by myself because I can’t hold something to get up because of my health status. So, it would be nice to see outside It’s frustrating to be lying down all the time
		P5	I’m free to do whatever I want But, now that the rules are a little bit more strict Now it’s a little bit uncomfortable…
		P14	You know, some of the patients are really mentally disturbed… Those people usually don’t think about other people. So, I wish the government would take care of that…
		P15	I don’t usually hang out with people… I don’t want to be around them because they’re weird. And I work out in the early morning. I’ve been doing it by myself for an hour in the morning since I first came here, so I can lie down and sit down because I’m tired afterward. I don’t want to go out, because I don’t like it

#### Acceptable privacy level

The five-point Likert scale revealed that certain participants were not satisfied with their acoustic and visual privacy. However, when prompted with open-ended questions about potential improvements, most participants did not provide detailed responses. Rather, they expressed that the privacy conditions provided by the facility were acceptable. Additionally, one participant (P6) said that a certain level of noise was acceptable because complete silence can cause anxiety in people, who are inherently social beings.

## Discussion

Overall, the findings of this study offer exploratory insights into enhancing the quality of life for residents in assisted living facilities. The results suggest that environmental design enhancements, such as lowering noise levels, offering more spacious personal rooms, and accommodating individual privacy needs, could improve sleep quality and boost resident satisfaction.

The results offer preliminary insights into sleep quality, homeliness, and privacy in assisted living facilities in South Korea. The data indicates that some residents suffer from poor sleep quality because of sensory disruptions, such as noise, light, and room temperature. This aligns with prior studies that identified the sensory environment as a crucial element in sleep disruption. For example, one study found that noise and light frequently affect the sleep quality of older adults in care facilities^28^. Regarding clinical implications, nursing staff can use this information to implement strategies aimed at minimizing these disruptions. For example, they can ensure that nighttime health checks are conducted as discreetly as possible to avoid disturbing residents’ sleep. Additionally, they can regulate room temperatures to maintain a comfortable sleeping environment for residents.

However, this study also highlights the distinct challenges encountered by individuals with health conditions residing in residential care. For example, regular health checks by staff, which often involve turning on lights, can disrupt their sleep. Beyond environmental factors, this study pinpointed the presence of staff, uncomfortable bedding, and anxiety as factors that may contribute to subpar sleep quality. These insights align with previous research showing that social and psychological elements affect sleep quality^29^ and stress levels^30^.

Concerning home-like environments, most participants were satisfied with the home-like environment features of the facility, with social support from staff and family, private rooms, and quality services. However, some participants were dissatisfied, recommending enhancements such as larger private rooms, increased personal space, improved neighborhood relations, and health condition-based resident separation.

The current findings, compared with previous literature, confirm the significance of social support from staff and loved ones in creating a home-like environment^31^. These results highlight the importance of staff training and fostering social interactions between residents and staff to strengthen residents’’ sense of belonging and connection within the facility. This study highlights the importance of creating a home-like atmosphere within long-term care facilities to promote residents’ well-being. Nursing staff can play a crucial role in fostering such environments by providing social support and personalized care to residents. They can engage residents in activities that evoke feelings of home, such as decorating their living spaces with personal items and organizing family visits. Moreover, staff can advocate for improvements in facility design, such as offering larger rooms and incorporating elements that resemble home furnishings.

Furthermore, “our finding that private rooms increase residents’’ satisfaction align with those of previous research^32,33^. Private rooms provide a sense of independence, control, and privacy, which can enhance well-being and quality of life. Therefore, long-term care facilities should consider offering private rooms to enhance residents’ satisfaction with their living environments.

The current study’s findings highlight the necessity for larger rooms, personal spaces, and improved neighborhood relations, echoing prior research^4^. These improvements can bolster residents’ sense of comfort, safety, and social connectedness, which are vital for a home-like environment. Moreover, this study confirms the significance of segregating residents by health condition, a practice supported by earlier studies emphasizing the need for personalized care^34^. Such segregation can improve residents’ quality of life, mitigate negative emotions, and increase satisfaction with their living environment.

Privacy is essential for quality care in long-term care facilities^4^. It involves an individual’s control over their personal information and the option to be alone when needed. The physical environment in long-term care facilities significantly affects residents’ privacy. Thus, participants were satisfied with their acoustic and visual privacy, likely because of effective soundproofing, strategic spatial layout, and the privacy features offered by the facility. While single rooms provide more privacy, two of the related challenges are loneliness and social isolation. To address this concern, long-term care facilities must proactively implement strategies to foster social connectedness and mitigate the risk of isolation among residents. While transitioning to more spacious or shared living arrangements may be a long-term solution, immediate steps can be taken to enhance social interaction within existing facilities. This may include organizing group activities, establishing community spaces for communal gatherings, facilitating peer support programs, and encouraging meaningful engagement between residents and staff. Additionally, leveraging technology and innovative communication platforms can provide opportunities for residents to stay connected with their loved ones and the broader community, thereby reducing feelings of loneliness and isolation.

However, notably, some participants expressed dissatisfaction with their privacy conditions, even though the overall satisfaction scores were high. This aligns with past research indicating that certain long-term care residents are dissatisfied with their privacy^35^. Specifically, dissatisfaction has been noted with shared bedrooms, the absence of privacy curtains, and poor sound insulation. According to Burack et al.^35^, participants expressed a desire for greater privacy and control over their living arrangements. They reported feeling uncomfortable with having a roommate and often felt they had no choice. Participants also expressed concerns about the staff entering their rooms without knocking or respecting their privacy. Similarly, in the present study, some participants wished for larger rooms, TVs in shared rooms, and improved soundproofing. Additionally, this study revealed that while most participants were satisfied with their privacy conditions, some expressed dissatisfaction and desired enhancements. Nursing staff can address these concerns by respecting residents’ privacy preferences and ensuring that their personal space is adequately protected. Staff should knock before entering residents’ rooms and provide them with the option to control their environment, such as adjusting curtains for visual privacy. Moreover, facilities can consider implementing measures to reduce noise levels and enhance soundproofing to further protect residents’ privacy.

Some participants in this study were unaffected by noise, potentially influencing their satisfaction with acoustic privacy. This finding highlights the importance of individual differences in privacy perception within long-term care facilities. This study suggests that although the physical environment is crucial for ensuring privacy, personal factors such as hearing impairment and social requirements are also key in shaping privacy perceptions.

Finally, this study is especially significant for societies with rapidly aging populations, such as South Korea. South Korea’s population in 2070 is expected to decrease by 27% compared with 2020, and the population aged 65 or older is expected to increase from 17.5 to 46.4%^36^. In response to one of the highest aging rates in the world, the number of long-term care facilities continues to increase. Hence, based on the results of this study, it is important to provide LTCUs that can improve the quality of life of older adults through a home-like environment.

### Limitations

The findings of this study should be interpreted with caution owing to several inherent limitations. First, the sample size, consisting of only 15 participants, raises concerns about the generalizability of the results to the broader population of older adults in LTCUs. The limited participant pool may not adequately represent the diversity and variability within this demographic, making it challenging to draw universally applicable conclusions. To enhance the robustness of future investigations, it is imperative to conduct studies with more extensive and more diverse samples.

Moreover, the ’study’s exclusive focus on a single LTCU in Seoul, South Korea, introduces geographical and contextual limitations. The characteristics and experiences of older adults in this particular LTCU may not be representative of those in different regions or countries, emphasizing the need for multi-site studies to capture a more comprehensive understanding of the subject matter.

A notable concern is the potential for self-selection bias, as participants were chosen based on their willingness to participate, even after being screened for health conditions and length of residence. This could introduce a bias towards individuals who are more inclined to engage in research activities, potentially skewing the results and limiting the generalizability of the findings to the broader population of older adults in LTCUs.

The following limitation of the study pertains to the sample selection process, which relied on screening by facility staff to exclude participants with cognitive issues. This approach may not have been adequate to accurately assess participants’ cognitive statuses, as it lacked formal inclusion/exclusion criteria specifically designed to evaluate cognitive functioning. Consequently, there is a possibility that individuals with mild cognitive impairments or undiagnosed cognitive issues may have been included in the study sample. Additionally, it is important to acknowledge that the study’s findings may be more relevant to cognitively intact assisted living residents rather than the entire resident population of long-term care facilities. This limitation suggests that the results may not fully capture the experiences and perspectives of residents with cognitive impairments, who may face unique challenges and have different care needs. Therefore, future research should consider implementing more rigorous methods for assessing cognitive status and ensure that the sample is representative of the broader population of residents in long-term care facilities.

Additionally, another limitation of this study is the absence of data collection regarding participants’ health statuses and frailty. This omission limits the depth of understanding regarding how residents’ health conditions may influence their perceptions of home-like features and privacy in long-term care settings. Future research could address this limitation by incorporating measures of health status and frailty into data collection procedures, allowing for a more comprehensive analysis of the factors influencing residents’ perceptions in long-term care environments.

Furthermore, while the study collected both qualitative and quantitative data using the GDS and PSQI, the predominant reliance on qualitative data hampers its ability to perform robust quantitative comparisons. This limitation restricts the statistical power of the study, hindering the establishment of statistically significant conclusions. Future research endeavors should aim to strike a balance between qualitative and quantitative methodologies to provide a more comprehensive understanding of the studied phenomena. In sum, the limitations identified in this study underscore the necessity for cautious interpretation and highlight areas for improvement in future research efforts.

Finally, this study lacks a comprehensive analysis regarding the environmental factors affecting older adults in LTCUs. While measurements of illumination, noise, temperature, and humidity were conducted, further analysis to determine the optimal environmental conditions for older adults was not included. Additionally, aligning these findings with the daily schedule and activities of older adults was not thoroughly explored. Future research should integrate such analyses to better understand the relationship between environmental factors and the well-being of older adults in long-term care settings, ultimately facilitating the creation of more conducive living environments tailored to their needs.

## Conclusion

This exploratory study in an LTCU sought to understand older adults’ perceptions of their living spaces, focusing on home-like features and privacy, both visual and acoustic. Employing qualitative methods–specifically semi-structured interviews–the study found that most participants were satisfied with the home-like atmosphere and privacy of their living environments. However, few participants voiced concerns about the adequacy of privacy conditions.

Most participants scored low on the GDS and PSQI. Sleep was primarily disrupted because of the sensory environment, staff interruptions, and room temperature. Most participants appreciated the social support from attentive staff and surroundings, particularly the privacy of their rooms and the distinct spatial arrangements. Nonetheless, some wished for larger rooms. This study offers valuable perspectives on older adults’ perceptions of their living spaces in LTCUs, with implications for enhancing their quality of life in long-term care.

## Data Availability

The datasets generated and/or analyzed during the current study are not publicly available due to confidentiality issues as a small number of participants included in this study and the facility does not wish to public the data, but are available from the corresponding author on reasonable request.
